# Complementary feeding and effect of spontaneous fermentation on anti-nutritional factors of selected cereal-based complementary foods

**DOI:** 10.1186/s12887-018-1369-3

**Published:** 2018-12-23

**Authors:** Degnet Teferi Asres, Amanuel Nana, Girma Nega

**Affiliations:** Department of Applied Human Nutrition, Biotechnology Research Institute, Bahir Dar Institute of Technology, Bahir Dar University, Bahir Dar, Ethiopia

**Keywords:** Fermentation, Complementary feeding, Antinutritional factors, Minerals contents

## Abstract

**Background:**

Malnutrition has been responsible directly or indirectly for 10.9 million deaths worldwide annually among children under five. Childhood malnutrition is highly related to poor nutritional quality diet in developing countries where there is limited access to animal based foods. Most foods consumed by young children are cereal based which contain high amounts of anti-nutritional factors. Fermentation is thought to significantly lower the content of anti-nutrients in cereal grains. This study therefore, aimed to determine complementary feeding practices and effect of spontaneous fermentation on anti-nutritional factors and mineral contents of selected cereals.

**Methods:**

Cross sectional survey was conducted in Ebinat district to determine complementary feeding practices among 324 lactating mothers. Laboratory analysis was carried out for teff and wheat cereal grains to determine the effect of spontaneous fermentation on anti-nutrients as well as mineral contents.

**Results:**

Prevalence of appropriate complementary feeding practice was 1.5%. Fermentation of the sampled cereals for 12 h significantly (*p* < 0.05) reduced total phytate and total tannin. The reduction continued and most of the reduction of phytate and tannin contents occurred during the 72 h of fermentation for both cereal samples. However, the reduction for some fermentation times was not statistically significant. A significant (*p* < 0.05) variation was also noticed in the total amounts of calcium, iron and zinc in both sampled cereals within the 72 h of fermentation.

**Conclusion:**

Prevalence of appropriate complementary feeding practice was very low. There were significant reductions of phytate and tannin contents with concomitant increments of minerals after fermentation of cereals. Phytate: mineral ratios were significantly decreased after fermentation for all the parameters examined. It is recommended to ferment cereals while preparing complementary foods for children so as to enhance their micronutrient uptake.

## Background

Childhood malnutrition continues to be one of the most stubborn public health problems throughout the developing world including Ethiopia. Demographic and Health surveys data from twenty-one developing countries indicated that poor complementary feeding of children aged 6–23 months contributes to negative growth trends [1]. In sub-Saharan African countries, suboptimal infant feeding practices including poor nutritional quality of complementary foods, micronutrient deficiencies accompanied by frequent infections contribute to high mortality rates among infants and young children [2].

Inappropriate complementary feeding practices remain one of the most determinant factors which prone children susceptible to irreversible outcomes of stunting, poor cognitive development and increased risk of infectious diseases [3]. For instance, malnutrition has been responsible directly or indirectly for 10.9 million (60%) of the annual children under five deaths worldwide [4].

Malnutrition is particularly a significant public health problem for infants and young children in Ethiopia. The Ethiopian mini demographic and health survey (DHS, 2014) shows that national rates of stunting, underweight and wasting among under 5 years children were 40, 25 and 9% respectively [5], have declined only slightly in the last decade and remained serious public health problems.

It is strongly evident that promotion of appropriate complementary feeding practices reduces the rates of stunting and contributes to better child health and growth outcomes [6]. For this reason, WHO and UNICEF recommend introduction of adequate complementary foods at 6th month and improving the quantity and quality of foods children consume while maintaining breastfeeding [7]. It is believed that practicing exclusive breastfeeding for the first six months, introduction of adequate complementary foods at 6th month and continued breastfeeding for 2 years of age or beyond have a potential role to improve the nutritional status of children in developing countries [8]. However, in Ethiopia, only 4% of young children at the age of 6–23 months are fed in accordance with recommended IYCF practices [5].

In developing countries there is limited access to animal products such as meat, egg, fish, milk and milk products even though they provide high concentrations of micronutrients including heme iron, calcium and zinc. The main dietary sources of iron, calcium and zinc in these countries are therefore cereals and legumes [9]. Nevertheless, cereal based foods contain high amounts of anti-nutritional factors which strongly bind minerals like iron, calcium and zinc [10] lead to poor bioavailability. On the other hand, large amounts of these nutrients are required during early life due to accelerated physical and mental growth [11], so ensuring their bioavailability is critical.

The selected district for the survey which is found in south Gondar administrative zone, Northwest Ethiopia is one of the most food insecure districts. It is also long known for its high level of vulnerability to food insecurity with more than one-third of the total population being undernourished [12]. As a result, it is more likely that children should have limited access to animal products and their diet should be cereal based that contain high amounts of anti-nutrients.

Fermentation is an important process which helps to lower the content of anti-nutrients (phytates, tannins, and polyphenols) of cereal grains [13]. It activates several endogenous enzymes and results in products with reduced anti-nutritional factors [14]. It also improves other nutrients and sanitary qualities of foods [15]. Fermentation of cereals generally leads to improvement in nutritional value, digestibility, shelf life, texture, taste and aroma of cereal products [16]. This study therefore, aimed to evaluate the effect of spontaneous fermentation on contents of anti-nutrients and bioavailability of minerals.

## Methods and materials

### Survey

A community-based cross-sectional survey was conducted in Ebinat district from May to July, 2016. It is one of the 12 districts of South Gondar administrative zone of Amhara region with an estimated area of 2494.27 km^2^ having 35 rural and 2 urban Kebeles administrations. The sample size for the survey was determined using a single population proportion formula considering the following assumptions: Proportion of appropriate complementary feeding (*p* = 10.75%) in Tigray region, Northern Ethiopia [17]; 95% level of confidence (Z = 1.96); marginal error (d = 5%)

$$ n=\frac{\mathrm{Z}\ \upalpha /2\Big)\ 2\mathrm{p}\ \left(1-\mathrm{p}\right)}{\mathrm{d}2\ } $$

From above formula, the calculated sample size was 147. By considering design effect of 2 and non-response rate of 10%, the final sample size was estimated to be 324.

The district was purposively selected due to its high level of vulnerability to food insecurity and has a very long history of aid [12]. In the district there are 37 kebeles of which 20% were randomly selected. Proportional to size allocation method was used to take appropriate sample from each kebele. Finally, systematic random sampling method was used to select respondent households. When more than one, mother-infant/child pairs were randomly selected.

Data for the survey were collected using a validated questionnaire adapted from the Ethiopian Health and Demographic Survey and WHO and LINKAGE project which were designed to assess infant and young child feeding practices in developing countries [4, 18, 19]. A structured questionnaire was used to collect data on maternal complementary feeding practices using four indicators; timely introduction of complementary feeding, minimum dietary diversity, minimum meal frequency and minimum acceptable diet. The indicators were operationalized as follows [20].

Timely introduction of complementary feeding: proportion of children at the age of 6–23 months who started complementary foods at 6th month.

Minimum dietary diversity: proportion of children at the age of 6–23 months who consumed foods from four or more food groups during the previous day. The seven food groups used for formulation of this indicator were: grains, roots and tubers; legumes and nuts; dairy products (milk, yogurt, cheese); flesh foods (meat, chicken and liver/organ meats); eggs; vitamin A rich fruits and vegetables; and other fruits and vegetables.

Minimum meal frequency: proportion of breastfed and non-breastfed children at the age of 6–23 months who got solid, semi-solid or soft foods the minimum number of times or more (2 times for breastfed infants 6–8 months; 3 times for breastfed children 9–23 months and 4 times for non-breastfed children 6–23 months) in the preceding day of the survey.

Minimum acceptable diet: proportion of breastfed children at the age of 6–23 months who had at least the minimum dietary diversity and the minimum meal frequency during the preceding day of the survey.

Appropriate complementary feeding practice: If the mother responds correctly all the above four indicators and if at least one indicator was not fulfilled, it was assumed to be inappropriate.

The data quality for the survey was ensured through training of data collectors, supervision and pre-test on 10% of the sample size. The questionnaire was prepared in English, translated in to Amharic and contextualized to the local situation. Pre-test was done before actual data collection. The completeness and consistency of the collected data have been checked before the study participants leave. The survey data were entered in to Epi Info version 6, checked for missing values and outliers and analyzed using SPSS (SPSS version 20).

### Laboratory analysis

Most commonly used cereals for complementary feeding were Teff (Kuncho) and wheat (LOGAWSHIBO; TT14) and collected from Amhara seed enterprise. In all the treatments (sample preparation, laboratory determination and data analysis), the cereals were processed separately.

Sample materials were cleaned manually to remove husks, damaged grains, stones, dust, light materials, glumes, stalks, undersized and immature grains and other extraneous materials.

The cleaned grains of each cereal were dried in drying oven at 55 ^ͦ^C for about 2 h to facilitate and make the milling process conducive. The dried cereals were milled into flour to pass through a 1 mm aperture size test sieve to obtain a fine powder. The milled samples were then packed in airtight polythene plastic bags until further analysis.

Suspensions of cereals flour in de-ionized water were prepared in plastic containers at a concentration of 1:3 dilutions (*w*/*v*). The cereals flour slurries were allowed to ferment spontaneously at room temperature (20–23 ^ͦ^C) for 0, 12, 24, 36, 48 and 72 h in 12 plastic containers. The supernatant was decanted and samples were withdrawn. The fermented samples were transferred to aluminum dishes after each fermentation time and dried in a hot air oven-drier at 70 °C for 36 h. All samples were analyzed for Phytate, tannin and minerals (Ca, Fe, and Zn).

Phytate was determined by the method of Latta and Eskin [21] which was later modified by Vantraub and Lapteva [22]. About 0.1000 g of fresh samples were extracted with 10 ml 2.4% HCl in a mechanical shaker for 1 h at an ambient temperature and centrifuged at 3000 rpm for 30 min. The clear supernatant was used for phytate estimation. A 2 ml of Wade reagent (containing 0.03% solution of FeCl_3_.6H_2_O and 0.3% of sulfosalicilic acid in water) was added to 3 ml of each sample solution and the mixture was mixed on a Vortex for 5 s. The absorbances of the sample solutions were measured at 500 nm using UV-VIS spectrophotometer. A series of standard solutions of phytic acid were prepared in 0.2 N HCl. A 3 ml of standard was added into 15 ml of centrifuge tubes with 3 ml of water which was used as a blank. A 1 ml of the Wade reagent was added to each test tube and the solution was mixed on a Vortex mixer for 5 s. Then mixtures were centrifuged for 10 min and the absorbances’ of the solutions (both the sample and standard) were measured at 500 nm by using de-ionized water as a blank. Standard curves were made from absorbance versus concentration and the slope and intercept was used for calculation. Phytate: mineral molar ratios were calculated using the molecular weight of IP6 = 660.

Tannin content was determined by the method of Burns [23] latter modified by Maxson and Rooney [24]. About 2.0 g of the samples were weighed in a screw cap test tube. The samples were extracted with 10 ml of 1% HCl in methanol for 24 h at room temperature with mechanical shaking and the solutions were centrifuged at 1000 rpm for 5 min after 24 h shaking. A 1 ml of supernatant was taken and mixed with 5 ml of vanillin-HCl reagent (prepared by mixing equal volume of 8% HCl in methanol and 4% vanillin in methanol). D-catechin was used as standard for condensed tannin determination. A 40 mg of D-catechin was weighed and dissolved in 1000 ml of 1% HCl in methanol and used as stock solution. A 0, 0.2, 0.4, 0.6, 0.8 and 1 ml of stock solutions were taken in test tube and the volume of each test tube was accustomed to 1 ml with 1% HCl in methanol. A 5 ml of vanillin-HCl reagent was added into each test tube. After 20 min, the absorbance of sample solutions and the standard solutions were measured at 500 nm by using water to zero the spectrophotometer. The calibration curves were made from the series of standard solutions using SPSS-20. Standard curves were prepared from absorbance versus concentration and the slopes and intercepts were used for calculation.

The mineral contents (calcium, iron, and zinc) were determined by the procedure of AOAC (1984) using an Atomic Absorption Spectrophotometer. After removal of organic material by dry ashing, the residue was dissolved in dilute acid. The solution was sprayed into the flame of Atomic Absorption Spectrophotometer and the absorption of the metal to be analyzed was measured at a specific wavelength. The stock standard solutions of minerals (iron, zinc and calcium) were diluted with 0.3 N HCl to concentrations that fall within the working range (0, 0.6, 1.0, 1.4, 1.8, μg/ml for zinc analysis; 1.0, 1.5, 2.5, and 3.0 μg/ml for calcium analysis and 0, 2.0, 6.0, 10.0 12.0 μg/ml for iron analysis). The ash obtained from dry ashing was mixed with 5 ml of 6 N HCl and dried on a low temperature hot plate. A 7 ml of 3 N HCl was added to the dried ash and heated on the hot plate until the solution just boils. The ash solution was cooled to room temperature at open air in a hood and filtered through a filter paper into a 50 ml graduated flask. A 5 ml of 3 N HCl was added into each crucible dishes and heated until the solution just boiled, cooled, and filtered into the flask. The crucible dishes were again washed three times with de-ionized water and the washings were filtered into the flask. A 2.5 mL of 10% Lanthanum chloride solution was added into each graduated flask. Then the solution was cooled and diluted to the mark (50 ml) with de-ionized water. A blank was prepared by taking the same procedure as the sample.

For all experiments, determinations were made in triplicates. Errors were calculated as standard deviations of the mean (SD) and SAS 9.1.3 service pack 4 was used to analyze the results. Means were separated using Duncan’s Multiple Range Test. Significance was accepted at 0.05 level of probability.

## Results

### Complementary feeding practices

Majority (70.1%) of the mothers initiated complementary feeding at 6 months. However, only 1.5% of mothers practiced appropriate complementary feeding (Table 1).

**Table 1:** Complementary feeding practice of mothers for their children, Ebinat district, Northwestern Ethiopia, 2016

Variables		Frequency (N)	Percentage (%)
Timely introduction of complementary feeding		227	70.1
Dietary diversity from 24 h recall	Grains, roots and tubers	172	53.1
	Legumes and nuts	107	33.0
	Dairy products	116	35.8
	Vitamin A-rich fruits	80	24.7
	Flesh foods	40	12.3
	Eggs	100	30.9
	Other fruits & vegetables	158	48.8
Minimum dietary diversity (≥4 food groups)		15	4.6
Minimum meal frequency		155	47.84
Minimum acceptable diet		10	3.08
Appropriate complementary feeding		5	1.5

### Household level food processing

During the preparation of family foods, mothers/care takers perform various household level food processing techniques. Out of all respondents, 196 (60.5%) reported that they usually ferment grains during the preparation of family foods (Table 2). However, they hardly do for complementary foods.

**Table 2:** Proportion of mothers/care takers considered household food processing techniques and willing in the production and marketing of complementary foods, Ebinat district, Northwest Ethiopia, 2016

Household food processing	Frequency(n)	Percentage (%)
Fermentation	196	60.5
Roasting	241	74.4
Soaking	191	59.0
Germination	171	52.8
Removal of bran	229	70.7
Willingness to use complementary foods produced at community level	315	97.2
Interest to be a member of women’s groups for production and marketing of complementary foods	279	86.1

### Production and marketing of complementary foods

Almost all (97.2%) of the respondents reported that they are willing to use complementary foods produced at community level if they got opportunity in their locality. The huge majority (86.1%) of them responded in favor of involvement in the production and marketing of complementary foods (Table 2).

### Effect of spontaneous fermentation on anti-nutrients and mineral contents

Phytate (mg/100 g) and tannin (mg/100 g) contents of teff and wheat samples (expressed as plus or minus of the standard deviations) are shown in Table 3 as affected by different periods of fermentation (0, 12, 24, 36, 48 and 72 h).

**Table 3:** Phytate and tannin content of teff and wheat samples during spontaneous fermentation, Ebinat district, Northwestern Ethiopia, 2016

Fermentation time	Parameters			
	Phytate(mg/100 g)		Tannin(mg/100 g)	
	Teff	Wheat	Teff	Wheat
0 h	541.45 ± 13.40a	464.10 ± 0.00a	0.87 ± 0.00a	0.58 ± 0.00a
12 h	471.84 ± 13.39b	417.69 ± 0.00b	0.79 ± 0.00b	0.56 ± 0.00b
24 h	448.63 ± 26.79c	394.49 ± 0.00c	0.78 ± 0.00c	0.54 ± 0.00c
36 h	464.10 ± 0.00bc	394.49 ± 0.00c	0.77 ± 0.00d	0.49 ± 0.00d
48 h	433.16 ± 26.79c	417.69 ± 0.00b	0.76 ± 0.00e	0.48 ± 0.00e
72 h	440.89 ± 23.20bc	371.28 ± 0.00d	0.74 ± 0.00f	0.47 ± 0.00f

Values are means of triplicate samples (± SD). Means not sharing a common letter in a column are significantly different at p < 0.05 as assessed by Duncan’s Multiple Range Test

Phytate contents of unfermented samples were 541.45 ± 13.40 and 464.10 ± 0.00 mg/100 g for teff and wheat, respectively while the tannin contents were 0.87 ± 0.00 and 0.58 ± 0.00 mg/100 g for the sample cereals respectively. Fermentation of the cereals for 12 h significantly (*p* < 0.05) reduced total phytate from 541.45 to 471.84 and 464.10 to 417.69 mg/100 g for teff and wheat, respectively (Table 3). The reduction rate continued (though some are not significant) and reached its minimum value of 440.89 mg/100 g and 371.28 mg/100 g for teff and wheat respectively when the flours were fermented for 72 h.

Fermentation of the sampled cereals for 12 h also significantly (p < 0.05) reduced total tannin from 0.87 to 0.79 and 0.58 to 0.56 mg/100 g for teff and wheat, respectively (Table 3). The reduction continued and reached its minimum value of 0.74 mg and 0.47 mg/100 g for teff and wheat cereals respectively when the flours were fermented for 72 h.

Furthermore, correlation analysis also showed that phytate and tannin contents are inversely related with period of fermentation with Pearson correlation coefficient (r) values of 0.771 and 0.858 for Phytate and tannin respectively for the teff sample. Phytate and tannin contents of wheat also inversely correlated with fermentation time with Pearson correlation coefficient (r) values of 0.816 and 0.948 for phytate and tannin respectively.

The regression model that estimates the relationship between phytate content with respect to period of fermentation was also Y = 504.10–1.17x and Y = 445.65–0.99x for teff and wheat, while the curve fit that relates fermentation time with tannin content in this study was Y = 0.83–0.02x and Y = 0.57–0.01x for these cereals respectively.

The mineral contents of the two sampled cereals (teff and wheat) are shown in Table 4. The values of the unfermented flour in both sampled cereals were significantly different (*p* < 0.05) from fermented samples in all the parameters along with period of fermentation time. The total mineral values of both spontaneously fermented sampled cereals were higher as compared to the unfermented samples of the cereals.

**Table 4:** Mineral contents of teff and wheat samples during spontaneous fermentation, Ebinat district, Northwestern Ethiopia, 2016

Fermentation time	Parameters					
	Ca (mg/100 g)		Fe(mg/100 g)		Zn(mg/100 g)	
	Teff	Wheat	Teff	Wheat	Teff	Wheat
0 h	160.00 ± 6.67^e^	80.00 ± 0.00^d^	6.19 ± 0.48^d^	3.10 ± 0.24^e^	1.93 ± 0.02^f^	1.15 ± 0.02^d^
12 h	266.67 ± 6.67^d^	86.67 ± 0.00^c^	10.79 ± 0.55^b^	4.92 ± 0.27^d^	2.01 ± 0.02^e^	1.26 ± 0.02^c^
24 h	282.22 ± 3.85^c^	86.67 ± 0.00^c^	10.95 ± 0.95^b^	5.24 ± 0.95^d^	2.11 ± 0.02^d^	1.41 ± 0.02^b^
36 h	291.11 ± 3.85^c^	86.67 ± 0.00^c^	9.68 ± 0.73^c^	6.51 ± 0.27c	2.28 ± 0.02^c^	1.22 ± 0.02^c^
48 h	304.44 ± 3.85^b^	97.78 ± 3.85^b^	11.43 ± 0.48^b^	8.33 ± 0.24 ^b^	2.48 ± 0.02^b^	1.37 ± 0.04^b^
72 h	320.00 ± 6.67^a^	110.00 ± 3.33^a^	15.94 ± 0.24^a^	13.10 ± 0.24^a^	2.63 ± 0.04^a^	1.51 ± 0.02^a^

Values are means of triplicate samples (± SD). Means not sharing a common letter in a column are significantly different at *p* < 0.05 as assessed by Duncan’s Multiple Range Test

The contents of calcium, iron and zinc in unfermented cereals were 160, 6.19 and 1.93 for teff and 80.0, 3.10 and 1.15, for wheat in mg/100 g respectively and increased in to 320, 15.94 and 2.63 for teff and 110.0, 13.10 and 1.51 for wheat after 72 h of fermentation.

Furthermore, the phytate to mineral molar ratios in the two sampled cereals (teff and wheat) are shown in Table 5. The phytate: calcium molar ratios were below 0.24 (critical molar ratio) in all the fermented and unfermented samples of teff and 72 h fermented wheat. The phytate: iron molar ratios were above > 0.15 (critical point) in both samples of cereals examined. Phytate: zinc molar ratios were also above 15 in both samples of cereals analyzed.

**Table 5:** Effect of spontaneous fermentation on molar ratios of phytate and minerals in teff and wheat samples, Ebinat district, Northwest Ethiopia, 2016

Fermentation time	Parameters					
	phytate: calcium		phytate: iron		phytate: zinc	
	Teff	Wheat	Teff	Wheat	Teff	Wheat
0 h	0.21 ± 0.01a	0.35 ± 0.00a	7.44 ± 0.43a	12.77 ± 0.99a	27.68 ± 0.87a	39.87 ± 0.70a
12 h	0.11 ± 0.00b	0.29 ± 0.00b	3.72 ± 0.25b	7.22 ± 0.39b	22.72 ± 0.56b	32.53 ± 0.53b
24 h	0.10 ± 0.01c	0.28 ± 0.00c	3.49 ± 0.38cb	6.54 ± 1.21cb	20.89 ± 1.36c	27.62 ± 0.41b
36 h	0.09 ± 0.00c	0.28 ± 0.00c	4.08 ± 0.30cb	2.56 ± 0.04c	20.04 ± 0.18c	31.76 ± 0.46c
48 h	0.09 ± 0.01 dc	0.26 ± 0.01d	3.22 ± 0.33c	5.45 ± 0.24d	17.27 ± 1.00d	30.01 ± 0.88d
72 h	0.08 ± 0.01d	0.21 ± 0.01e	2.35 ± 0.11d	3.78 ± 0.11e	16.48 ± 0.89d	24.17 ± 0.33e

Values are means of triplicate samples (± SD). Means not sharing a common letter in a column are significantly different at *p* < 0.05 as assessed by Duncan’s Multiple Range Test

## Discussion

In this study, the overall prevalence of appropriate complementary feeding practice and the effect of spontaneous fermentation on anti-nutrients and mineral contents were assessed. The phytate to mineral molar ratios were also presented.

### Complementary feeding practices

Timely introduction of complementary feeding was determined and possible provisions of most common non-human milk alternative liquids including water, milk, butter and fenugreek before the age of 6 months were also assessed*.* The result indicated that majority (70.1%) of the mothers initiated complementary feeding at 6 months though low compared to WHO cut-off point (80 to 94%) for good practice of complementary feeding [19] and reports in India, 77.5% [25] and Abyi-Adi, Ethiopia, 79.7% [17], while it is consistent with a study from Bangladesh, 71% [26] and Benishangul Gumuz Regional State, Northwest, Ethiopia, 73.9% [27]. However, it was higher than the findings (65.7%) reported by a review in Southern Ethiopia [28], 63% in Lalibela district, Northeast Ethiopia [29] and 56.4% in Enemay district, Northwest Ethiopia [30]. The discrepancies might be attributed to differences in status of institutional delivery, health care settings and utilization of antenatal care (ANC) and postnatal care (PNC) services as nutrition education and counseling are components of these services that would bring an added benefit to improve mothers’ awareness on appropriate child feeding practices [17]. In this study, many women met the criteria for introduction of complementary foods at 6 months and this might be attributed to the effort of health extension workers as mothers who had sufficient knowledge on complementary feeding practices are more likely to introduce timely as compared to mothers who had not sufficient knowledge [30].

Proportion of mothers offered four or more food groups to their children was far low (4.6%) compared to similar studies in India, 15.2% [31], Kenya, 17.9% [32], Zambia, 12% [33],and Ethiopian national report, 10.8% [34], northern Ethiopia, 17.8% [17], Arsi Negele district, southeastern Ethiopia [35] that reported 18.8% and Dangila, Northwest, Ethiopia which reported 12.6% [36]. The discrepancies might be attributed to differences in agroecological characteristics as the study district is drought prone area that might result in low accessibility to diversified foods. The most frequently consumed food groups were grains, roots and tubers (53.1%) while the least frequently consumed were animal source foods such as meat and fish (12.3%). Only 30.9% of children consumed eggs and 35.8% got dairy products. This might be an indication that the area has limited access to animal source foods and children might be more likely to suffer from micronutrient deficiencies.

Proportion of mothers who met the minimum meal frequency is lower (47.8%) compared to other reports such as Paskistan, 56.4% [37], northern Ghana, 57.3% [38] and other similar studies like Arsi Negele district, southeastern Ethiopia, 67.3% [35] and Dabat District, northwest Ethiopia, 72.2% [39]. However, it is similar with a study from Dangila, Northwest, Ethiopia, 50.4% [28]. The discrepancies might be due to differences in literacy level, awareness of mothers on IYCF and other socioeconomic statuses as this study was in remote district area.

Over all practice of appropriate complementary feeding is very low (1.5%) compared with other similar studies including a review report from five Asian counties [40], Tanzania [41], Ethiopian national average [18], Enemay district, Northwest Ethiopia, [30] and Arsi Negele district, southeastern Ethiopia [35]. These differences might be attributed to the district’s vulnerability to drought that would result in limited access to diversified foods which is implicated in very low diet diversity.

### Effect of spontaneous fermentation on anti-nutrients and mineral contents

Fermentation of the sampled cereals for 12 h significantly (*p* < 0.05) reduced total phytate in both teff and wheat sample cereals. The results of this study are in agreement with other similar studies [42, 43], which reported significantly low concentration of phytate sorghum produces compared to unfermented one. A study from Nigeria also concluded that fermentation is the most effective processing technique that reduced phytic acid in the cereal flours [44].

It has been suggested that the loss of phytate during fermentation could be a result of the activity of native phytase and the fermentative microflora as reported by different researchers [42, 45, 46]. In this study, most of the reduction in phytate occurred during the 72 h of fermentation. This might be due to the prevailing pH which is considered to be an optimum for microbial phytase activity since all enzymes have a specific pH in which they function most capably [42]. However, for some of the fermentation times reductions in phytate content for both cereals were not significant. These fluctuations in values of phytate content could be attributed to the metabolic activities of the microorganisms.

Fermentation of the cereals for 12 h also significantly (*p* < 0.05) reduced total tannin in both cereals. The reduction continued and reached its minimum value when the flours were fermented for 72 h. Overall reduction in tannin content of fermented samples is significant (*p* < 0.05) compared to unfermented samples in all fermentation times. It is in agreement with a study where reduction in tannin content of fermented samples of sorghum was highly significant (*P* < 0.05) compared to unfermented samples [42]. Reduction in tannin contents due to fermentation might have been caused by the activity of polyphenoloxidase or tanniase of fermenting microflora [44]. However, this result is in contrary to other study that reported fermentation for 36 h at room temperature was found to cause no changes in tannin content of fermented dough for millet cultivars [45]. Other study also reported that, tannin content of pearl millet sample showed significant increase after fermentation [47]. This might be attributed to differences in room temperature where microorganisms could suitably grow and produce tannin degrading enzymes.

The mineral contents of the unfermented flours in both teff and wheat sampled cereals were significantly different (*p* < 0.05) from fermented samples along with period of fermentation time. Overall, a significant (p < 0.05) variation was noticed in the total amounts of calcium, iron and zinc in both sampled cereals within the 72 h of fermentation. This is in agreement with a study conducted on soymilk where there was increase in calcium, magnesium, zinc and iron contents during natural fermentation of soymilk [48]. The increase in the mineral contents of fermented samples may be due to the fact that minerals (bound to anti-nutrients) were released from chelated complex compound through the activities of microorganisms responsible for the fermentation [49]. However, there were reductions in the mineral contents during fermentation times of 24 h and 36 h. The reduction of total minerals in those fermentation times might be ascribed due to the microorganisms could have utilized some of the hydrolyzed elements for their metabolic activities as the time might be comfortable for their metabolic actives [50]. On the other hand, the results of the present study contradict with the observation made on maize that fermentation does not have effect on the contents of total minerals [51].

Furthermore, the phytate to mineral molar ratios in the two cereals (teff and wheat) were also assessed. The phytate:calcium molar ratio was below the critical molar ratio [52] in all the fermented and unfermented samples of teff and 72 h fermented wheat. Thus, the result indicates favorable Ca absorption in teff and 72 h fermented wheat.

The bioavailability of iron was low since phytate:iron molar ratios were above the critical value [53] in both fermented and unfermented cereals examined which is regarded as indicative of poor iron bioavailability. This might be because of the high levels of phytic acid in both the samples of cereals examined.

Phytate:zinc molar ratios were also > 15 in both the sample cereals analyzed which it is an indicative of poor zinc bioavailability [53–55].

In general, phytate: mineral ratios were significantly decreased after fermentation for all the parameters examined even if the critical values were not achieved for iron and zinc. This study is in agreement with those of fermented rice-dehulled black gram blends [56] and fermented cereal based complementary foods [57, 58]. The lower phytate: mineral ratios for the fermented teff and wheat cereal grains might be partly ascribed to the decreased content of phytic acid during fermentation. Thus, fermentation enhances bioavailability of minerals by degrading phytate with microbial and native phytases that entangle macro- and trace elements. Above mentioned studies also indicated that fermentation hydrolyzed anti-nutrients from their organic bonds to increase mineral bioavailability.

### Limitations of the study

Due to limitations in finance and long distance of the survey area from the laboratory, sensory analysis of fermented products had not been carried out with survey participants.

## Conclusions

Prevalence of appropriate complementary feeding practice was very low which indicated the need of immediate support and follow up. There were significant reductions of phytate and tannin contents with concomitant increments of minerals after fermentation of cereals. Phytate: mineral ratios were significantly decreased after fermentation for all the parameters examined. Besides of spontaneous fermentation lowers phytic acid and tannin contents and improve the extractability of minerals, it is a promising and simple method as it doesn’t require even fuel. Therefore, spontaneous fermentation should be promoted with community awareness interventions through existing health and agriculture system of the government so as to enhance micronutrient uptake of young children. Health and agriculture extension workers should be trained so as to include it in their package. Community and religious leaders should also be educated so as to get better acceptance by the community. To this effect, the information should be disseminated to the regional bureaus as well as district offices.

## Abbreviations

AOAC: Association of Official Analytical Chemists
DHS: Demographic and Health Survey
IYCF: Infant and Young Child Feeding
SAS: Statistical Analysis System
SD: Standard Deviation
SPSS: Statistical Package for Social Sciences
UNICEF: United Nations for Children’s Fund
WHO: World Health Organization
